# The Influence of Metabolic Syndrome on the Development of Gastrointestinal Malignant Tumors—A Review

**DOI:** 10.3390/medicina61061025

**Published:** 2025-05-31

**Authors:** Vesna Brzački, Andrija Rančić, Snežana Tešić Rajković, Ivan Nagorni, Marko Stamenković, Elena Stanković, Nikola Milutinović, Aleksandar Vukadinović

**Affiliations:** 1 Clinic for Gastroenterohepatology, University Clinical Center of Niš, 18000 Niš, Serbia; brzackiv@gmail.com (V.B.); snezanatesic@yahoo.com (S.T.R.); ivannagorni@gmail.com (I.N.); drmarkostamenkovic@gmail.com (M.S.); stankovicelena008@gmail.com (E.S.); 2 Clinic for Abdominal Surgery, University Clinical Center of Niš, 18000 Niš, Serbia; milutinac88@gmail.com (N.M.); acavuk89@gmail.com (A.V.)

**Keywords:** metabolic syndrome, gastrointestinal malignant tumors, diabetes mellitus, obesity

## Abstract

*Background and Objectives*: Metabolic syndrome (MetS) is characterized by a cluster of metabolic abnormalities, including abdominal obesity, hyperglycemia, insulin resistance, dyslipidemia, and hypertension. Growing evidence suggests that these components may contribute to the development of gastrointestinal (GI) malignancies. This review aims to explore the association between MetS and GI cancers, including esophageal, gastric, pancreatic, and colorectal cancers. *Materials and Methods*: A narrative literature review was conducted using PubMed, incorporating 22 sources published between 1991 and 2024. Search terms included “gastrointestinal malignant tumors”, “metabolic syndrome”, “diabetes mellitus”, and “obesity”. Priority was given to large-scale studies from Europe, America, and Asia. Case reports, commentaries, and conference abstracts were excluded. *Results*: By analyzing the available literature data, this study determined that hyperinsulinemia (IGF-1 pathway), hyperglycemia, and obesity (>102 cm in men and >88 cm in women) are highly associated with the development of esophageal cancer (primarily with Barret’s long and short segment as precancerosis), gastric cancer (through reactive oxygen species), and both pancreatic (1.5–2.4 higher risk) and colorectal cancer (30% higher risk). Patients with a high BMI (>40 kg/m^2^) show a 20%- or 1.18-times greater risk of developing colorectal cancer and a 1.72-times higher risk of developing pancreatic cancer. There is not enough evidence on the specific influence of hypertriglyceridemia, low HDL cholesterol, and high blood pressure on the development of gastrointestinal malignancy. However, those three conditions have shown a low to moderate association (from 6% to 12%) with the development of colorectal cancer. *Conclusions*: Metabolic syndrome (MetS) is increasingly being recognized as a significant risk factor for the development and progression of gastrointestinal cancers. Key components such as obesity, hyperglycemia, insulin resistance, and type 2 diabetes mellitus appear to contribute to carcinogenesis through mechanisms involving chronic inflammation, oxidative stress, and immune dysregulation. Further research is needed to clarify the biological pathways linking MetS to gastrointestinal malignancies and to inform effective prevention strategies.

## 1. Introduction

Metabolic syndrome (MetS) is a multifactorial disorder characterized by a cluster of interconnected metabolic abnormalities. Components of MetS typically include abdominal obesity, insulin resistance, impaired glucose metabolism, hypertension, and atherogenic dyslipidemia manifested by elevated triglyceride levels and low high-density lipoprotein (HDL) cholesterol. This syndrome reflects a state of systemic metabolic dysfunction and increases the risk of cardiovascular disease, type 2 diabetes mellitus, and several malignancies [1].

The Adult Treatment Panel III (ATP III) of the National Cholesterol Education Program (NCEP) has provided one of the most widely used clinical definitions of MetS [2]. According to the ATP III criteria, MetS is diagnosed when an individual exhibits at least three out of five of the following risk factors:

1. An increased waist circumference (abdominal obesity): >102 cm in men and >88 cm in women;
2. Hypertriglyceridemia: a fasting triglyceride level of >1.7 mmol/L (150 mg/dL);
3. Low HDL cholesterol: <1.03 mmol/L (40 mg/dL) in men and <1.29 mmol/L (50 mg/dL) in women;
4. An elevated blood pressure: systolic ≥130 mmHg and/or diastolic ≥85 mmHg, or current use of antihypertensive medication;
5. Impaired fasting glucose: ≥6.1 mmol/L (110 mg/dL) or use of anti-diabetic medication.

This diagnostic framework has served as the foundation for numerous epidemiological and clinical investigations. Although subsequent revisions by the International Diabetes Federation (IDF) and the American Heart Association/National Heart, Lung, and Blood Institute (AHA/NHLBI) have adjusted some of these parameters, particularly regarding ethnicity-specific waist circumference cut-offs, the core concept of MetS remains consistent [2].

Beyond its well-established cardiovascular implications, an increasing body of evidence suggests that MetS is also a significant risk factor for the development and progression of gastrointestinal malignancies. The proposed pathophysiological links between MetS and carcinogenesis include chronic systemic inflammation, increased oxidative stress, insulin resistance, hyperinsulinemia, and the dysregulation of growth-promoting pathways such as the insulin-like growth factor-1 (IGF-1) axis. These processes can facilitate tumor initiation, progression, and resistance to apoptosis [3].

Recent studies have demonstrated a compelling association between MetS and an elevated risk of cancers of the esophagus, stomach, pancreas, colon, and rectum. Obesity and an increased body mass index (BMI) have been independently associated with gastrointestinal cancer development. It has been estimated that obesity may contribute to approximately 20% of cancer-related deaths in women and 14% in men. The overall mortality risk is increased by 83% in individuals with three or more MetS components [4,5]. Furthermore, emerging data indicate that MetS is associated with a higher risk of gastric cancer, especially in East Asian populations, such as Korea and China, where the recent adoption of Western dietary habits has been accompanied by rising obesity and MetS prevalence [6,7].

Gender-specific associations have also been observed. Colorectal cancer risk appears to be more closely linked with MetS in women [8], whereas pancreatic cancer demonstrates a stronger correlation in men [8,9]. Moreover, individual components of MetS, such as hyperglycemia, hyperinsulinemia, and visceral adiposity, have been implicated in the pathogenesis of esophageal adenocarcinoma, particularly in the setting of Barrett’s esophagus [10]. These findings suggest that the components of MetS may not contribute equally to cancer risk, and their synergistic effects may vary by cancer type, sex, and ethnicity. Nevertheless, the evidence consistently points toward a substantial role of metabolic dysregulation in gastrointestinal oncogenesis.

Given the growing global burden of obesity and metabolic syndrome, understanding the connections between MetS and cancer is of great importance. The early identification of high-risk individuals and the implementation of lifestyle and pharmacologic interventions such as weight loss, improved glycemic control, and the use of insulin-sensitizing agents may offer promising strategies for cancer prevention. This narrative review aims to synthesize current knowledge on the association between MetS and gastrointestinal cancers, highlight potential mechanistic pathways, and identify gaps in the literature that warrant further research.

## 2. Materials and Methods

To discover the connection between MetS and gastrointestinal malignancy, the literature from the PubMed database was examined. This was conducted using a combination of search terms such as “gastrointestinal malignant tumors”, “metabolic syndrome”, “diabetes mellitus”, and “obesity”, starting from the beginning of 1991 until the end of 2024. Our narrative review included information from around 22 articles, including original and review articles, meta-analyses, and statistical data. The point of interest was larger population studies, mostly focused on European, American, and Asian populations. Commentaries, case reports, and conference abstracts were excluded.

Due to the complex and not well-known connection between gastrointestinal malignant tumors and components of metabolic syndrome, this review was written as a narrative literature review. The aim was to provide insight into potential molecular mechanisms that can lead to metabolic syndrome, and also to further estimate the possible cancer development risk in patients with MetS.

## 3. Common Molecular Mechanisms in Malignancy Development

### 3.1. Adipose Tissue and Cancer

Adipose tissue plays a significant role in the pathogenesis of MetS. It is often considered to offer a lot more than just the storage of lipids. As a whole endocrine organ, adipose tissue can secrete a large amount of mediators and modulate different signaling cascades. There are functional, immunological, and structural support cells in adipose tissue. Also, adipose blood vessels supply nutrients and oxygen and further spread inflammatory mediators around the body [11].

This is why obesity is considered a chronic, low-grade inflammatory state. A lot of mediators are responsible for this inflammation. There is an increased secretion of tumor necrosis factor alpha (TNF-α), interleukin-6 (IL-6), plasminogen activator inhibitor (PAI-1), monocyte chemotactic protein-1 (MCP-1), leptin, and visfatin [12]. Also, levels of adiponectin production and peroxisome proliferator-activated receptor gamma are decreased [12,13]. Lipolysis in adipose tissue is highly stimulated, resulting in an increase in free fatty acids. These fatty acids can reach the liver, where they are involved in the synthesis of very-low-density lipoproteins (VLDLs). This state of dyslipidemia is followed by a dramatic reduction in high-density lipoproteins (HDLs) and a further increase in low-density lipoproteins (LDLs). Many studies have found that all these components can further induce oxidative stress, angiogenesis, and insulin resistance, which can lead to the development of a specific cancer [5,14].

### 3.2. Insulin Resistance and Cancer

Insulin resistance occupies a significant role in pathogenesis [13]. Among the two main subtypes of insulin receptors (IR-A and IR-B), IR-B is responsible for intracellular signaling. There is a possibility that this effect is partly responsible for cancer development [15,16]. Many studies suggest that the main pathway of insulin-induced cancerogenesis is the overexpression of insulin growth factor-1 (IGF-1). The production of IGF-1 in the liver is primarily induced by growth hormone. On the other hand, one of the main roles of insulin is in the upregulation of growth hormone receptors. This can lead to IGF-1 overstimulation and the overexpression of IGF-1 receptors. IGF-1 receptor (IGF-1R) is expressed in almost all cells and is homologous to IR. This allows for the formation of heterodimers or hybrid receptors (IGF-1R/IR) that regulate further survival and cell proliferation [17]. The stimulation of these hybrid receptors can induce a very complex molecular cascade reaction. This includes the activation of β tyrosine kinase, phosphoinositide-3-kinase, and protein kinase B and the inhibition of Bcl-2-associated death promoter. The final result is reduced apoptosis and the promotion of further cellular growth, resulting in the formation of cancer [15,17]. This pathway is found to be more common in colon cancer [18].

### 3.3. Other Molecular Mechanisms of MetS and Cancer

As previously mentioned, leptin is a hormone secreted in adipose tissue. It plays an important role in basal metabolism increases and is responsible for satiety sensation. People with obesity have hyperleptinemia and leptin resistance [12,15]. Leptin can induce the secretion of vascular endothelial growth factor (VEGF) by activating nuclear factor-κB (NF-κB). VEGF leads to neo-angiogenesis, which is necessary for tumor growth [12]. Some studies have found that leptin can also induce cancer cell expansion through several signaling pathways [19]. A higher leptin level is associated with colon cancer and can contribute to colonic metastasis [15,19]. Unlike leptin, adiponectin levels are low in obese patients [20]. This hormone is also secreted in adipose tissue and has important anti-proliferative and anti-inflammatory effects [21]. It can also induce endothelial cell apoptosis and the inhibition of NF-κB. It is believed that lower adiponectin levels correlate with gastric cancer risk [22,23].

Similarly, cyclooxygenase-2 (COX-2) can increase neo-angiogenesis and the production of prostaglandins. This enzyme can inhibit apoptosis, which leads to faster tumor cell invasion and growth. COX-2 is overexpressed in pancreatic cancer [24]. More cytokines like interleukins IL-1β, IL-6, IL-8, and IL-10 and macrophage inflammatory protein-1 are in focus as potential triggers for tumor growth. Inflammation is related to gastric, pancreatic, esophageal, and colorectal cancer [25].

## 4. Association Between MetS and Common Gastrointestinal Malignancies

### 4.1. Association Between MetS and Esophageal and Gastric Cancer

Esophageal and gastric cancers are globally among the most common and deadly malignancies. Esophageal cancer (EC) ranks seventh, while gastric cancer (GC) ranks as the fourth-most common cancer worldwide [26,27]. In 2020, the International Agency for Research on Cancer (IARC) reported approximately 1.1 million new cases of gastric cancer and about 604,000 new cases of esophageal cancer worldwide [27]. The highest incidence has been noted in Japan, China, South America, Eastern Asia, and Eastern Europe, while the lowest is in Africa [28]. In South Korea, the 5-year survival rate for esophageal cancer has improved significantly, rising from 12.1% in 1993–1995 to 34.6% in 2009–2013. This improvement arises from early detection and efficient treatment strategies [29]. Regarding GC, Japan boasts a high 5-year survival rate for gastric cancer, reaching up to 90%, due to advanced screening programs. In contrast, European countries report a 5-year survival rate ranging from 10% to 30%, reflecting differences in healthcare infrastructure and early detection capabilities [30].

Over the years, many nonspecific risk factors have been connected to EC and GC. It is well known that alcohol consumption, smoking, and low fruit intake can induce certain types of EC, such as esophageal squamous cell carcinoma (ESCC). Gastroesophageal reflux disease is considered the main risk factor for esophageal adenocarcinoma (EAC) [31]. Regarding GC, increased alcohol intake is especially associated with the development of non-cardiac types of GC. Additionally, in 1994, The World Health Organization described *Helicobacter pylori* (*H. pylori*) as a first-class carcinogen for the development of GC [32]. Although all of these risk factors are important, they can only explain around 46% of ESSC in the Asian population and around 76% of EAC in Australia [31,33].

Different risk factors can induce tumors, including components of MetS. Concerning EAC, one cohort and one case–control study found a positive link between metabolic syndrome and EAC risk. However, two other studies reported no association between Mets and any type of EC [34]. This discrepancy may arise from the studied population, particularly in Europe, where ESCC incidence is lower. In 2022, Lee Eun Ji and colleagues investigated the link between MetS and esophageal cancer in the Asian population, where ESCC is predominant [35]. In a sample of 6000 cases, Mets patients had an 11% higher risk of developing esophageal cancer than those without MetS, specifically for ESCC, as EAC is less common in Asia [29,35]. The unique biochemical environment may contribute to the higher esophageal cancer occurrences in MetS patients. Factors such as hyperinsulinemia, hyperglycemia, visceral adiposity, estrogen signaling, and IGF-1 pathways, alongside active inflammation, could trigger carcinogenesis and cancer progression, potentially explaining the MetS–EAC link [36]. A large prospective cohort study in America involving 900,000 adults examined the relationship between cancer and obesity, including MetS risk factors like smoking, inactivity, alcohol use, and diet. This study found a significant rise in esophageal, colorectal, and pancreatic cancer mortality. The most obese participants (BMI > 40) had higher cancer death rates, at 52% for males and 62% for females [37]. A smaller study of 102 patients linked MetS with Barrett’s esophagus (BE), finding that 46% of BE patients had MetS and 78% were obese. Furthermore, 60% of patients with long-segment Barrett’s esophagus (LSBE) had MetS, with 92% being centrally obese compared to those with short-segment BE. LSBE was associated with higher IL-6 levels and hyperinsulinemia than short-segment BE [38]. While a connection between metabolic syndrome and esophageal cancer exists, it remains unclear.

Regarding gastric cancer (GC), there is little evidence of the connection between MetS and the risk of gastric cancer development, except for some larger studies conducted in the Asian population [39]. A study conducted by Huang Dan and colleagues found that there was an increased risk of GC with higher glucose values, especially in women with lower values of HDL cholesterol [7]. These results correlate with a big Japanese study which reported that higher values of fasting plasma glucose significantly increase the risk of developing gastric cancer. A similar cohort study conducted in Norway on more than 192 thousand adults reported an increased GC risk in individuals with MetS. Also, European multicohorts with more than 564 thousand adults reported that hyperglycemia, hypertriglyceridemia, and low HDL cholesterol increased GC risk [40].

Although previous studies suggest that MetS often affects cancerogenesis in women, a study conducted by Lin Yulan and coworkers found that MetS was associated with gastric adenocarcinoma in both women and men. This study found that participants with a high waist circumference had around a 50% higher risk of developing gastric adenocarcinoma. Also, there was strong evidence of a connection between gastric adenocarcinoma of the cardia and abdominal obesity. Regarding obesity, the Korean Multi-Center Cohort study found a significant association between BMI and GC risk development only in *H. pylori*-uninfected adults [41,42]. MetS may contribute to GC development as a synergistic promoting factor. Several mechanisms that could result in GC genesis together are obesity, insulin resistance, IGF-1, angiogenesis, adipokines, and higher estrogen values. Higher secretion of insulin and IGF-1 due to insulin resistance also increases the production of reactive oxygen species. This oxidative stress could cause DNA damage, which further leads to changes in tumor suppressor genes and oncogenes [43]. There is an influence of MetS on GC development, mostly in the Asian population. Many studies suggest that MetS may be a modifiable GC risk factor. The examined association between metabolic syndrome and the development of EC and GC is shown in Table 1.

### 4.2. Association Between MetS and Pancreatic Cancer

Pancreatic cancer (PC) is the 12th most common malignant tumor today [44]. There has been around a 2.3-times higher incidence of PC over the past 30 years, and it could become the second leading cause of cancer-related deaths by 2030. This disease is more common in elderly men and rarely occurs before the age of 40 [45]. It has a five-year survival rate of around 10%, and it is the sixth-most common cause of cancer-related deaths in the United States. Although some risk factors, such as male gender, older age, chronic pancreatitis, diabetes mellitus, obesity, and cigarette and alcohol consumption, are connected to PC, its exact etiology remains unclear [46,47]. Identifying modifiable risk factors for PC should be prioritized to reduce the burden of pancreatic cancer worldwide.

As in other cancers, there is evidence that associates MetS components with the development of pancreatic cancer. A big meta-analysis based on four cohort studies and one case–control study found a 55% higher risk of pancreatic cancer development in individuals with MetS. This association was stronger in females than in males [9]. Probably one of the main risk factors for PC development and its consequences is diabetes mellitus. UK Biobank data suggests that PC risk is higher in adults with hyperglycemia and abdominal obesity [48]. Patients with longer-standing diabetes (longer than 3 years) have a 1.5–2.4-times higher risk of developing PC. Other studies have concluded that patients who suffer from diabetes mellitus for over 20 years also remain at an elevated risk of pancreatic cancer [49]. Mayo Clinic reported that in their study on over 2000 adults, around 1% of patients with newly diagnosed diabetes mellitus developed PC within the first 3 years. According to a similar majority of studies, PC risk correlates with hyperglycemia and lower HDL cholesterol levels [50]. Also, similar results were presented in a Chinese cohort study, which concluded that an increase in blood glucose level by 1 mmol/L also increases the risk of PC development by 15%.

Excluding diabetes mellitus, some studies suggest that there is a significant association between BMI, waist circumference, and the risk of developing PC. A study by Aune D. found that there is an increased risk of pancreatic cancer development even among individuals within the normal BMI range [51,52]. A big study by Michaud and colleagues conducted on more than 46 thousand men and more than 117 thousand women concluded that adults with a BMI higher than 30 kg/m^2^ had a 1.72-times higher risk of developing PC. Another Danish study conducted on over 293 thousand people, of whom 1268 had PC, reported similar results [53,54]. Differently, a study from 2020 conducted on over 112 thousand women and over 46 thousand men found that major weight loss in adulthood can increase the chance of PC development. Regarding previous research on the connection between MetS and PC, various molecular pathways were found. Some of those, including modulations in IGF-1 and IGF-2 responses and higher secretion of insulin and cytokines, could result in PC. Table 2. Those molecules can boost cell proliferation and angiogenesis and inhibit cell death. Also, reactive oxygen species play a big, yet not well-known role in the development of PC [55,56].

### 4.3. Association Between MetS and Colorectal Cancer

Colorectal cancer (CRC) is the third-most common malignant tumor and cause of cancer-related deaths globally today [57]. Its incidence has been increasing since the 1990s and today is estimated at around 1.4 million people, which represents 9.8% of all malignant tumors. The highest incidence is noted in Asia (52.3%) and Europe (26.9%), while the lowest is in Africa (3.4%) [58]. Many meta-analyses and systematic reviews have reported that MetS is associated with an approximately 1.3-times increased risk of CRC in both genders, but the risk in women is slightly higher. Also, the prevalence in young adults is around 7% globally, and this increases with age [9]. Colorectal carcinogenesis is a process that includes several of the following stages: the transformation from a normal epithelium and adenoma through to cancer.

Research on the association between MetS, BMI, and waist circumference on one side, and colorectal adenomas and cancer on the other, is more frequent today due to the higher incidence of these diseases. But these research results are pretty contradictory. Some studies have indicated an increased risk of adenomas and colorectal cancer, especially in white women and in patients with MetS [59]. This incidence is higher in obese women, but not in obese men. However, in a study that included just obese men, there was a higher incidence of colorectal adenoma development [60]. A higher BMI and obesity may increase the chance of adenomas and further cancer development by increasing the mitosis of adenoma cells in the early phase [61]. A big Canadian community-based study from 2020 studied the influence of obesity and MetS on the prevalence of colorectal adenoma. It concluded that the risk of CRC development increases with age and in male smokers, and that a higher BMI has a strong influence on cancer development [62]. Also, one meta-analysis found that an increase in BMI of 5 kg/m^2^ in men gives a 1.24-times higher relative risk of CRC. Similar results in women cannot be concluded due to differences in fat distribution [63]. The European Prospective Investigation into Cancer and Nutrition showed that central obesity is an equally strong risk factor for CRC in both males and females, but body weight and BMI are associated with a higher CRC risk only in males [64].

Therefore, CRC may be highly related to an increased abdominal circumference and central obesity. A study conducted by Katherine Esposito and colleagues found a 29% higher risk of CRC development in patients with impaired fasting glucose, impaired glucose tolerance, and diabetes mellitus. This correlates with a bigger meta-analysis which showed a 30% higher risk of CRC among diabetic patients. Recent studies have found that the risk of CRC rises in patients with hypertension (up to 9%), high triglyceride levels (up to 6%), and lower HDL cholesterol levels (up to 12%). Although these components of MetS are important, each of them has a lower influence on CRC development regarding fully manifested MetS. A previously mentioned study by Katherine Esposito and coworkers also did not find a significant correlation between hypertension and CRC or adenoma. This study also did not find any connection between high or low levels of HDL-cholesterol and CRC risk [9]. But a recent European study consisting of 1238 patients revealed that there is a decreased risk of CRC in patients with higher levels of HDL-cholesterol [65].

It is documented that MetS and its components also influence cancer-related deaths. One study reported that a higher BMI (over 30 kg/m^2^) is associated with a 1.75- and 1.25-times higher relative risk of cancer death in both men and women [66]. These results correlate with a big meta-analysis from 2007 which included 31 studies and over 70 thousand colorectal cancers. In this study, individuals with a BMI over 30 kg/m^2^ had a 20% higher risk of developing CRC than individuals with a normal body weight. Also, the risk of developing CRC increased by 7% for every 2 kg/m^2^ increase in BMI. Similarly, the risk of CRC development increased by 4% for every 2 cm increase in waist circumference [67]. A big Korean cohort study concluded that BMI has a strong influence on CRC development, and that the risk of developing cancer is higher in the distal colon and rectum than in the proximal colon [68]. Differently, some recent systematic reviews have found that male individuals with a higher BMI have a 1.18-times greater risk of developing colon cancer than rectal cancer. The possible reason for the gender difference in the association between BMI and CRC could be explained by the different hormonal levels in women, especially estrogen. Finally, the Women’s Health Initiative Estrogen Plus Progestin trial showed a 37% reduction in the incidence of invasive CRC in postmenopausal women who were using hormones [69]. This studied CRC development is shown in Table 3.

## 5. Limitations and Strengths

In conclusion, chronic inflammation, altered cytokine profiles, and immune dysregulation commonly observed in obesity, type 2 diabetes mellitus, and hyperglycemic states may serve as potential central mechanisms in tumor initiation and progression.

Given these associations, early intervention through lifestyle modifications such as weight reduction, adherence to a balanced diet, and the pharmacologic management of hyperglycemia (e.g., metformin) may offer potential in reducing cancer risk in the general population. Although discrepancies exist across studies, the available data support a substantial role of MetS in the pathophysiology of gastrointestinal cancers.

Our study is not without limitations. A limitation of this narrative review is the limited number of patients, because there is no available data for certain types of gastrointestinal malignancies in some populations. Additionally, our review focused only on surveyed populations from Europe, Asia (mainly Korea), and America and Canada, excluding the populations of Africa and Australia. Also, we could not include information on the exact molecular mechanism of carcinogenesis caused by MetS components. Finally, our data is based on claims data and does not include information on all histologic subtypes.

Further research is warranted to elucidate the precise biological mechanisms linking MetS with gastrointestinal oncogenesis and to explore targeted preventive and therapeutic strategies. A clearer understanding of these pathways will enhance our knowledge of cancer etiology beyond the established environmental and genetic risk factors. Also, an adequate screening program for carcinomas would represent an important step in primary prevention, the discovery of possible mechanisms of carcinogenesis, and the detection of malignant tumors at an early stage. The discovery of the main metabolic mechanisms involved simultaneously in the development of malignancy and metabolic syndrome could be a main point for the development of future targeted therapy.

## 6. Conclusions

There is growing evidence supporting a significant association between metabolic syndrome (MetS) and the development of gastrointestinal malignancies, including esophageal, gastric, pancreatic, and colorectal cancers. Key components of MetS, such as hyperinsulinemia (via the IGF-1 signaling pathway), hyperglycemia, central obesity, and elevated body mass index (BMI), have demonstrated a strong link with increased cancer risk. Specifically, individuals with abdominal obesity (waist circumference >102 cm in men and >88 cm in women) and those with a BMI of >40 kg/m^2^ exhibit a markedly higher risk of developing colorectal and pancreatic cancers. Moreover, Barrett’s esophagus, both long and short segments, has been implicated as a precancerous condition closely associated with hyperinsulinemic states and central obesity, facilitating the progression to esophageal adenocarcinoma. Similarly, in gastric carcinogenesis, hyperglycemia appears to contribute through mechanisms involving increased oxidative stress.

While the individual contributions of hypertriglyceridemia, low HDL cholesterol, and elevated blood pressure remain less clearly defined, current evidence suggests a modest association ranging from 6% to 12% with colorectal cancer risk. This indicates that not all components of MetS exert the same degree of influence on carcinogenesis, underscoring the need for a nuanced understanding of their individual and synergistic effects.

## Figures and Tables

**Table 1:** Association between MetS components and esophageal and gastric cancer development.

MetS Components	Esophageal Cancer	Gastric Cancer
Hyperinsulinemia (IGF-1 pathway)	Moderate to high association (primarily with Barrett’s long and short segment) [36]	Moderate to high association (through reactive oxygen species) [43]
Obese (>102 cm in men, >88 cm in women)	Highly associated (form 78–92%) [38]	Highly associated (about 50% higher risk) [41]
BMI (> 40 kg/m^2^)	Moderate association (52% in male, 62% in female) [37]	Moderate association (primarily in adenocarcinoma) [41,42]
Hypertriglyceridemia Low HDL cholesterol	No confirmed association No confirmed association	Potential association (primarily in postmenopausal period) [7]
High blood pressure	No confirmed association	No confirmed association

**Table 2:** Association between MetS components and pancreatic cancer development.

MetS Components	Pancreatic Cancer
Hyperglycemia (Diabetes mellitus)	Moderate to high association (1.5–2.4 higher risk) [49]
Obese and BMI (>30 kg/m^2^)	Moderate association (1.72 higher risk) [51,52,53,54]
Hyperinsulinemia (IGF-1 pathway)	Potential association [55,56]
Hypertriglyceridemia Low HDL cholesterol	No confirmed association No confirmed association
High blood pressure	No confirmed association

**Table 3:** Association between MetS components and colorectal cancer development.

MetS Components	Colorectal Cancer
Hyperglycemia (Diabetes mellitus)	High association (30% higher risk) [9,66,69]
Obese and BMI (>30 kg/m^2^)	High association (20% or 1.18 times higher risk) [64,66,67]
Hypertriglyceridemia Low HDL cholesterol	Low to moderate association (up to 6%) [9] Moderate association (up to 12%) [9,65]
High blood pressure	Low to moderate association (up to 9%) [9]

## Data Availability

The data presented in this study are available on request from the corresponding author.

## Abbreviations

MetS	Metabolic syndrome
BMI	Body mass index
TNF-α	Tumor necrosis factor alpha
IL-6	Interleukin-6
PAI-1	Plasminogen activator inhibitor
MCP-1	Monocyte chemotactic protein-1
IGF-1	Insulin growth factor-1
VEGF	Vascular endothelial growth factor
NF-κB	Nuclear factor-κB
EC	Esophageal cancer
EAC	Esophageal adenocarcinoma
ESCC	Esophageal squamous cell carcinoma
BE	Barrett’s esophagus
LSBE	Long-segment Barrett’s
GC	Gastric cancer
PC	Pancreatic cancer
CRC	Colorectal cancer

## References

[1] Alberti K., Zimmet P., Shaw J. (2005). Metabolic syndrome—A new worldwide definition. Lancet.

[2] DeFronzo R.A., Ferrannini E. (1991). Insulin resistance. A multifaceted syndrome responsible for NIDDM, obesity, hypertension, dyslipidemia, and atherosclerotic cardiovascular disease. Diabetes Care.

[3] Calle E.E., Rodriguez C., Walker-Thurmond K., Michael J.T. (2003). Overweight, obesity, and mortality from cancer in a prospectively studied cohort of U.S. adults. N. Engl. J. Med..

[4] Jaggers J.R., Sui X., Hooker S.P., LaMonte M.J., Matthews C.E., Hand G.A., Blair S.N. (2009). Metabolic syndrome and risk of cancer mortality in men. Eur. J. Cancer.

[5] Cowey S., Hardy R.W. (2006). The metabolic syndrome: A high-risk state for cancer?. Am. J. Pathol..

[6] Bray F., Ferlay J., Soerjomataram I., Siegel R.L., Torre L.A., Jemal A. (2018). Global cancer statistics 2018: GLOBOCAN estimates of incidence and mortality worldwide for 36 cancers in 185 countries. CA Cancer J. Clin..

[7] Huang D., Shin W.K., De la Torre K., Lee H.-W., Min S., Shin A., Lee J.-K., Kang D. (2023). Association between metabolic syndrome and gastric cancer risk: Results from the Health Examinees Study. Gastric Cancer.

[8] Russo A., Autelitano M., Bisanti L. (2008). Metabolic syndrome and cancer risk. Eur. J. Cancer.

[9] Esposito K., Chiodini P., Colao A., Lenzi A., Giugliano D. (2012). Metabolic syndrome and risk of cancer: A systematic review and meta-analysis. Diabetes Care.

[10] Feng R.M., Zong Y.N., Cao S.M., Xu R.-H. (2019). Current cancer situation in China: Good or bad, news from the 2018 global cancer Statistics?. Cancer Commun..

[11] Ouchi N., Parker J.L., Lugus J.J., Walsh K. (2011). Adipokines in inflammation and metabolic disease. Nat. Rev. Immunol..

[12] Aballay L.R., Eynard A.R., Díaz M.D.P., Navarro A., Munoz S.E. (2013). Overweight and obesity: A review of their relationship to metabolic syndrome, cardiovascular disease, and cancer in South America. Nutr. Rev..

[13] Ye J. (2009). Emerging role of adipose tissue hypoxia in obesity and insulin resistance. Int. J. Obes..

[14] Eckel R.H., Alberti K.G.M.M., Grundy S.M., Zimmet P.Z. (2010). The metabolic syndrome. Lancet.

[15] Braun S., Bitton-Worms K., LeRoith D. (2011). The link between the metabolic syndrome and cancer. Int. J. Biol. Sci..

[16] Petridou E., Skalkidou A., Dessypris N., Moustaki M., Matzoros C., Spanos E., Trichopoulos D. (2001). Insulin-like growth factor binding protein-3 predicts survival from acute childhood leukemia. Oncology.

[17] Arcidiacono B., Iiritano S., Nocera A., Possidente K., Nevolo M.T., Ventura V., Foti D., Chiefari E., Brunetti A. (2012). Insulin resistance and cancer risk: An overview of the pathogenetic mechanisms. Exp. Diabetes Res..

[18] Giovannucci E. (2001). Insulin, insulin-like growth factors and colon cancer: A review of the evidence. J. Nutr..

[19] Moon H.S., Dalamaga M., Kim S.Y., Polyzot S.A., Hamnvik O.-P., Magkos F., Paruthi J., Mantzoros C.S. (2013). Leptin’s role in lipodystrophic and nonlipodystrophic insulin-resistant and diabetic individuals. Endocr. Rev..

[20] Kadowaki T., Yamauchi T. (2005). Adiponectin and adiponectin receptors. Endocr. Rev..

[21] Dalamaga M., Diakopoulos K.N., Mantzoros C.S. (2012). The role of adiponectin in cancer: A review of current evidence. Endocr. Rev..

[22] Pais R., Silaghi H., Silaghi A.C., Rusu M.C., Dumitrascu D.L. (2009). Metabolic syndrome and risk of subsequent colorectal cancer. World J. Gastroenterol..

[23] Ishikawa M., Kitayama J., Kazama S., Hiramatsu T., Hatano K., Nagawa H. (2005). Plasma adiponectin and gastric cancer. Clin. Cancer Res..

[24] Xu X.C. (2002). COX-2 inhibitors in cancer treatment and prevention, a recent development. Anticancer Drugs.

[25] Richardson V.R., Smith K.A., Carter A.M. (2013). Adipose tissue inflammation: Feeding the development of type 2 diabetes mellitus. Immunobiology.

[26] Ferlay J., Shin H.R., Bray F., Forman D., Mathers C., Parkin D.M. (2010). Estimates of worldwide burden of cancer in 2008: GLOBOCAN 2008. Int. J. Cancer.

[27] Morgan E., Arnold M., Camargo M.C., Gini A., Kunzmann A.T., Matsuda T. (2022). The current and future incidence and mortality of gastric cancer in 185 countries, 2020–2040: A population-based modelling study. EClinicalMedicine.

[28] Huang J., Koulaouzidis A., Marlicz W., Lok V., Chu C., Ho Ngai C., Zhang L., Chen P., Wang S., Yuan J. (2021). Global burden, risk factors, and trends of esophageal cancer: An analysis of cancer registries from 48 countries. Cancers.

[29] Shin A., Won Y.J., Jung H.K., Kong H.J., Jung K.W., Oh C.M., Choe S., Lee J. (2018). Trends in incidence and survival of esophageal cancer in Korea: Analysis of the Korea Central Cancer Registry database. J. Gastroenterol. Hepatol..

[30] Stock M., Otto F. (2005). Gene deregulation in gastric cancer. Gene.

[31] Marabotto E., Pellegatta G., Sheijani A.D., Ziola S., Zentilin P., De Marzo M.G., Giannini E.G., Ghisa M., Barberio B., Scarpa M. (2021). Prevention Strategies for Esophageal Cancer-An expert review. Cancers.

[32] Ishaq S., Nunn L. (2015). Helicobacter pylori and gastric cancer: A state of the art review. Gastroenterol. Hepatol. Bed Bench.

[33] Olsen C.M., Pandeya N., Green A., Webb P.M., Whiteman D.C. (2011). Population attributable fractions of adenocarcinoma of the esophagus and gastroesophageal junction. Am. J. Epidemiol..

[34] Drahos J., Ricker W., Pfeiffer R.M., Cook M.B. (2017). Metabolic syndrome and risk of esophageal adenocarcinoma in elderly patients in the United States: An analysis of SEER-Medicare data. Cancer.

[35] Lee J.E., Han K., Yoo J., Yeo Y., Cho I.Y., Cho B., Park J.-H., Shin D.W., Cho J.H., Park Y.-M. (2022). Association Between Metabolic Syndrome and Risk of Esophageal Cancer: A Nationwide Population-Based Study. Cancer Epidemiol. Biomark. Prev..

[36] Micucci C., Valli D., Matacchione G., Catalano A. (2016). Current perspectives between metabolic syndrome and cancer. Oncotarget.

[37] Tanaka T., Kawabata K., Sugie S. (2017). 4-Nitroquinoline 1-oxide-induced tongue and esophagus carcinogenesis in obese and diabetic TSOD mice. World J. Oncol..

[38] Ryan A.M., Healy L.A., Power D.G., Byrne M., Murphy S., Byrne P.J. (2008). Barrett esophagus: Prevalence of central adiposity, metabolic syndrome, and a proinflammatory state. Ann. Surg..

[39] Hu D., Peng F., Lin X., Chen G., Zhang H., Liang B. (2017). Preoperative metabolic syndrome is predictive of significant gastric cancer mortality after gastrectomy: The Fujian Prospective Investigation of Cancer (FIESTA) Study. EbioMedicine.

[40] Lin Y., Ness-Jensen E., Hveem K., Lagergren J., Lu Y. (2015). Metabolic syndrome and esophageal and gastric cancer. Cancer Causes Control.

[41] Lim J.H., Shin C.M., Han K., Yoo J., Jin E.H., Choi Y.J. (2022). Nationwide cohort study: Cholesterol level is inversely related with the risk of gastric cancer among postmenopausal women. Gastric Cancer.

[42] Jang J., Cho E.J., Hwang Y., Weiderpass E., Ahn C., Choi J. (2019). Association between body mass index and gastric cancer risk according to effect modification by *Helicobacter pylori* infection. Cancer Res. Treat..

[43] Simone S., Gorin Y., Velagapudi C., Abboud H.E., Habib S.L. (2008). Mechanism of oxidative DNA damage in diabetes: Tuberin inactivation and downregulation of DNA repair enzyme 8-oxo-7,8-dihydro-2′-deoxyguanosine-DNA glycosylase. Diabetes.

[44] Cai J., Chen H., Lu M., Zhang Y., Lu B., You L., Zhang T., Dai M., Zhao Y. (2021). Advances in the epidemiology of pancreatic cancer: Trends, risk factors, screening, and prognosis. Cancer Lett..

[45] GBD 2017 Pancreatic Cancer Collaborators (2019). The global, regional, and national burden of pancreatic cancer and its attributable risk factors in 195 countries and territories, 1990–2017: A systematic analysis for the Global Burden of Disease Study 2017. Lancet Gastroenterol. Hepatol..

[46] Chen W., Zheng R., Baade P.D., Zhang S., Zeng H., Bray F. (2016). Cancer statistics in China, 2015. CA Cancer J. Clin..

[47] Rawla P., Sunkara T., Gaduputi V. (2019). Epidemiology of Pancreatic Cancer: Global Trends, Etiology and Risk Factors. World J. Oncol..

[48] Xia B., He Q., Pan Y., Gao F., Liu A., Tang Y. (2020). Metabolic syndrome and risk of pancreatic cancer: A population-based prospective cohort study. Int. J. Cancer.

[49] Everhart J., Wright D. (1995). Diabetes mellitus as a risk factor for pancreatic cancer. A meta-analysis. JAMA.

[50] Chari S.T., Leibson C.L., Rabe K.G., Ramsom J., De Andrade M., Petersen G.M. (2005). Probability of pancreatic cancer following diabetes: A population-based study. Gastroenterology.

[51] Pang Y., Kartsonaki C., Guo Y., Bragg F., Yang L., Bian Z. (2017). Diabetes, plasma glucose and incidence of pancreatic cancer: A prospective study of 0.5 million Chinese adults and a meta-analysis of 22 cohort studies. Int. J. Cancer.

[52] Aune D., Greenwood D.C., Chan D.S.M., Vieira A.R., Navarro Rosenblatt D.A., Cade J.E. (2012). Body Mass Index, Abdominal Fatness and Pancreatic Cancer Risk: A Systematic Review and Non-Linear Dose-Response Meta-Analysis of Prospective Studies. Ann. Oncol..

[53] Michaud D.S., Giovannucci E., Willett W.C., Colditz G.A., Stampfer M.J., Fuchs C.S. (2001). Physical activity, obesity, height, and the risk of pancreatic cancer. JAMA.

[54] Nogueira L., Stolzenberg-Solomon R., Gamborg M., Sorensen T.I.A., Baker J.L. (2017). Childhood body mass index and risk of adult pancreatic cancer. Curr. Dev. Nutr..

[55] Yuan C., Babic A., Khalaf N., Nowak J.A., Brais L.K., Rubinson D.A. (2020). Diabetes, weight change, and pancreatic cancer risk. JAMA Oncol..

[56] Bastard J.P., Maachi M., Lagathu C., Kim M.J., Caron M., Vidal H. (2006). Recent advances in the relationship between obesity, inflammation, and insulin resistance. Eur. Cytokine Netw..

[57] Siegel R.L., Miller K.D., Goding Sauer A., Fedewa S.A., Butterly L.F., Anderson J.C. (2020). Colorectal cancer statistics, 2020. CA Cancer J. Clin..

[58] Sung H., Ferlay J., Siegel R.L., Laversanne M., Soerjomataram I., Jemal A. (2020). Global Cancer Statistics 2020: GLOBOCAN Estimates of Incidence and Mortality Worldwide for 36 Cancers in 185 Countries. A Cancer J. Clin..

[59] Stocks T., Lukanova A., Bjorne T., Ulmer H., Manjer J., Almquist M. (2010). Metabolic factors and the risk of colorectal cancer in 580,000 men and women in the metabolic syndrome and cancer project (Me-Can). Cancer.

[60] Neugut A.I., Garbowski G.C., Lee W.C., Murray T., Nieves J.W., Forde K.A. (1993). Dietary risk factors for the incidence and recurrence of colorectal adenomatous polyps. A case-control study. Ann. Intern. Med..

[61] Bardou M., Barkun A.N., Martel M. (2013). Obesity and colorectal cancer. Gut.

[62] Shapero T.F., Chen G.I., Devlin T., Gibbs A., Murray I.C., Tran S. (2017). Obesity Increases Prevalence of Colonic Adenomas at Screening Colonoscopy: A Canadian Community-Based Study. Can. J. Gastroenterol. Hepatol..

[63] Kim H., Apio C., Ko Y., Han K., Goo W., Heo G. (2020). Which National Factors Are Most Influential in the Spread of COVID-19?. Int. J. Environ. Res. Public Health.

[64] Pischon T., Lahmann P.H., Boeing H., Friedenreich C., Norat T., Tjonneland A. (2006). Body size and risk of colon and rectal cancer in the European Prospective Investigation Into Cancer and Nutrition (EPIC). J. Natl. Cancer Inst..

[65] Van Duijnhoven F.J.B., Bueno-De-Mesquita H.B., Calligaro M., Jenab M., Pischon T., Jansen E.H.J.M. (2011). Blood lipid and lipoprotein concentrations and colorectal cancer risk in the European Prospective Investigation into Cancer and Nutrition. Gut.

[66] Murphy T.K., Calle E.E., Rodriguez C., Kahn H.S., Thun M.J. (2020). Body Mass Index and Colon Cancer Mortality in a Large Prospective Study. Am. J. Epidemiol..

[67] Moghaddam A.A., Woodward M., Huxley R. (2007). Obesity and risk of colorectal cancer: A meta-analysis of 31 studies with 70,000 events. Cancer Epidemiol. Biomark. Prev..

[68] Kasprzak A. (2022). Role of the Ghrelin System in Colorectal Cancer. Int. J. Mol. Sci..

[69] Rossouw J.E., Anderson G.L., Prentice R.L., LaCroix A.Z., Kooperberg C., Stefanick M.L. (2002). Risks and Benefits of Estrogen Plus Progestin in Healthy Postmenopausal Women: Principal Results from the Women’s Health Initiative Randomized Controlled Trial. JAMA.

